# Hy-Bridge: A Hybrid Blockchain for Privacy-Preserving and Trustful Energy Transactions in Internet-of-Things Platforms

**DOI:** 10.3390/s20030928

**Published:** 2020-02-10

**Authors:** Mahdi Daghmehchi Firoozjaei, Ali Ghorbani, Hyoungshick Kim, JaeSeung Song

**Affiliations:** 1 Canadian Institute for Cybersecurity, University of New Brunswick, Fredericton, NB E3B 5A3, Canada; m.daghmechi@unb.ca (M.D.F.); ghorbani@unb.ca (A.G.); 2 Department of Electrical and Computer Engineering, Sungkyunkwan University, Suwon 164419, Korea; hyoung@skku.edu; 3 Department of Computer and Information Security, Sejong University, Seoul 05006, Korea

**Keywords:** Blockchain, Internet of things (IoT), Privacy-preserving, Credit-sharing

## Abstract

In the current centralized IoT ecosystems, all financial transactions are routed through IoT platform providers. The security and privacy issues are inevitable with an untrusted or compromised IoT platform provider. To address these issues, we propose Hy-Bridge, a hybrid blockchain-based billing and charging framework. In Hy-Bridge, the IoT platform provider plays no proxy role, and IoT users can securely and efficiently share a credit with other users. The trustful end-to-end functionality of blockchain helps us to provide accountability and reliability features in IoT transactions. Furthermore, with the blockchain-distributed consensus, we provide a credit-sharing feature for IoT users in the energy and utility market. To provide this feature, we introduce a local block framework for service management in the credit-sharing group. To preserve the IoT users’ privacy and avoid any information leakage to the main blockchain, an interconnection position, called bridge, is introduced to isolate IoT users’ peer-to-peer transactions and link the main blockchain to its subnetwork blockchain(s) in a hybrid model. To this end, a *k*-anonymity protection is performed on the bridge. To evaluate the performance of the introduced hybrid blockchain-based billing and charging, we simulated the energy use case scenario using Hy-Bridge. Our simulation results show that Hy-Bridge could protect user privacy with an acceptable level of information loss and CPU and memory usage.

## 1. Introduction

Nowadays, energy systems are becoming increasingly decentralized, diverse, and distributed. The increasing number of applications for renewable (e.g., solar or wind power) and distributed energy resources (DERs) has led to more energy being generated and consumed locally in microgrids, which is changing conventional centralized energy supply systems [1]. In a smart grid network, a new environment is provided to make DERs (e.g., active consumers) actively participate in power generation and consumption, by exploiting telecommunication techniques for control and monitoring [2,3,4]. The blockchain technology promises to enable energy transactions to be organized and tracked securely in a decentralized solution. With the use of a blockchain, consumers’ activities in the smart grid can be secured and recorded into immutable, transparent, and tamper-proof smart contracts [5].

Traditionally, the relationship in the energy market has been one-sided between an energy provider and consumers. Consumers use the energy from the grid and pay the bills issued by the provider, and the cycle repeats. The trend toward DERs changes this traditional relationship. For instance, consumers who have rooftop solar systems need bilateral energy relationships with the providers. Moreover, peer-to-peer (P2P) energy transactions between neighbors are a viable option and make the energy relationship multi-sided and more complicated. Consequently, when connected to the public grid, surplus solar energy can be delivered to the grid or sold to neighbors in microgrids. In the implementation of this scenario, consumers’ privacy is considered a significant issue similar to trustworthiness or reliability because of the multi-side relationship in the smart grid [6].

The increasing number of devices in the Internet-of-Things (IoT) would benefit modern power systems in a smart grid. In the smart grid, IoT applications collect data to indicate the need to purchase more energy or the ability to sell the surplus energy to neighbors [7]. Because of the emergence of such applications in the energy and utility industry, providing an infrastructure to manage the billing and charging functions is an essential functionality for the increasing number of different features. Furthermore, because of the lack of trust in providing standards, there is a need for a reliability mechanism in the IoT ecosystem. As IoT devices become more connected, more data will be shared and this might compromise the users’ privacy [8]. Normally, IoT platform providers act as middlemen to provide common services such as enforcing policies, controlling access, providing charging mechanisms, and billing. The key limitations in such centralized solutions are the low transparency and the single point of failure, where the trusted IoT platform provider can be targeted and compromised by the attacker, including a malicious insider [9,10]. Furthermore, IoT users’ privacy could be violated by an untrusted IoT platform provider with the basic monitoring capability. The drawbacks of a single point of failure and the impact of centralized authority with the potential risk of transaction data modification can pose security, trust, and privacy issues to IoT users in an untrusted network.

Therefore, efficient billing and payment capabilities are needed to address the trust and privacy issues in the multi-side energy relationships in the IoT energy sector. The impressive abilities of the blockchain technology in the area of trust, cost, flexibility, and reliability are considered to be promising for the finance sector. In this paper, we discuss how the blockchain technology can be practically applied to the IoT service layer to provide billing and charging functionalities to the IoT energy sector. We introduce Hy-Bridge, a hybrid architecture of blockchain developed on the IoT service layer. Developing the blockchain concept on the IoT service layer helps us introduce a compatible framework using blockchain-based billing and charging functionalities that can be adopted in various IoT domains, including a smart grid, which is composed of different IoT platforms from different vendors.

Hy-Bridge is a hybrid blockchain with subnetworks. It is designed to preserve IoT users’ privacy and provides a trustable workflow for billing and charging transactions; i.e., P2P energy transactions between users are not monitored. The privacy of IoT users is protected by separating the transactions of the power grid from those of the microgrid(s) in this hybrid framework. To this end, we introduce a bridge, an interconnection position between the main blockchain and its subnetwork(s). Hy-Bridge has a novel credit-sharing feature in that an IoT user can share an energy service with other users. This allows IoT users’ P2P energy transactions to be exchanged privately in the microgrid, as they are controlled by a subnetwork blockchain. To protect IoT users’ privacy, we perform *k*-anonymity protection in the service layer. *k*-Anonymity approaches are targeted at the applications that do not demand a true or pseudo-identity [11]. In the credit-sharing group, a *k*-anonymity group of IoT users is set and they can anonymously access to the shared energy service and control the activities of their IoT devices in the subnetwork blockchain. We introduce a local block in the subnetwork to handle the P2P transactions of the credit-sharing group in the microgrid. The local block has an additional header called credit header, to authorize IoT devices and enforce the credit-sharing policy [12]. Moreover, IoT users’ private data in the microgrid usage dataset are anonymized and masked into a generalized dataset available in the main grid. Therefore, the details of energy use and P2P transactions in the microgrid, which may lead to user identification and user profiling, are not leaked to the upper-layer entities of the smart grid.

To demonstrate the feasibility of the proposed framework, we consider a smart building with a rooftop solar power collection system. The solar power is shared among tenants (e.g., IoT users) through a local P2P energy network, i.e., a microgrid. In addition to the rooftop solar power system, the building uses the public power grid as another power source. It is assumed that the solar power system is owned by a service provider and that the property manager purchases all the solar energy and sells the surplus power to the tenants through the microgrid. Hy-Bridge provides trustful multi-sided relationships among the property manager, IoT users in the building, IoT platform provider, and the solar power service provider.

The main contributions of this article are as follows:

- We propose Hy-Bridge to preserve IoT users’ privacy in the billing and charging platform for the energy sector. In this hybrid blockchain, an interconnection position called the bridge, is introduced to link the main blockchain to its subnetwork(s). The bridge manages the transactions in two different blockchains to avoid user profiling and user identification. To this end, the bridge not only isolates all the P2P energy transactions of the microgrid but also provides anonymization protection to preserve IoT users’ privacy.
- We introduce the credit-sharing feature for IoT energy markets in the service layer. The concept of a local block with an additional header is introduced to handle the P2P transactions of the credit-sharing group in the microgrid. The credit header is used to authorize devices and enforce the credit-sharing policy.
- To analyze the performance of the introduced hybrid blockchain-enabled IoT architecture, we performed a simulation to evaluate the privacy leakage of Hy-Bridge. We analyzed the information loss in terms of *k*-anonymity. Finally, we measured the resource (memory and CPU) usage caused by Hy-Bridge to manage the power consumption transactions.

The rest of this paper is organized as follows. In Section 2, we describe the challenges and the need to have trustful transactions in a distrusted network. Section 3 introduces the related work. We briefly present an overview of blockchain and *k*-anonymity privacy protection in Section 4. In Section 5, the preliminaries of Hy-Bridge are explained and some use cases of Hy-Bridge are introduced in Section 6. In Section 7, we explain how Hy-bridge works and manages transactions. Its trustworthiness, privacy-preserving feature, and performance are analyzed in Section 8. Finally, we provide our conclusions and discuss future work in Section 9.

## 2. Trustworthy Challenges with Financial Transactions for IoT Platforms

**Figure 1:**
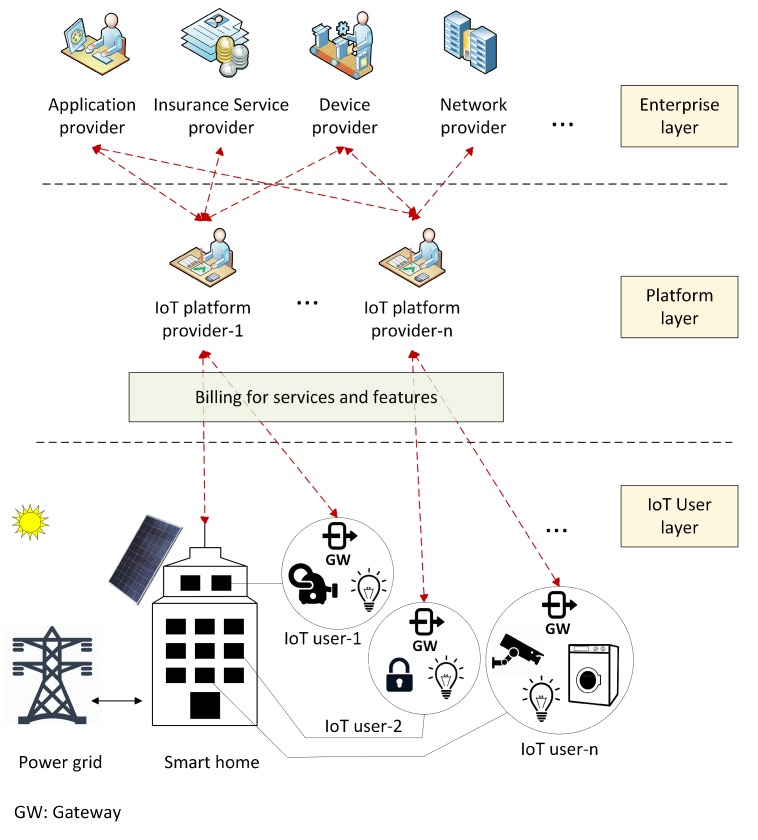
Financial relationships in Internet-of-Things (IoT) energy and utility market.

In general, the IoT ecosystem can be divided into three layers, namely the IoT user layer, the platform layer, and the enterprise layer. The user layer consists of physical components (e.g., IoT devices, gateways, and network connections); the platform layer provides tools for all aspects of the IoT platforms, such as data flow tools, stream processing, data storage, and external access. The enterprise layer is a collection of business applications and consists of service management technologies. As shown in Figure 1, an IoT platform provider has contracts with the third-party service providers in the enterprise layer and is the payment provider where IoT users and service providers have accounts. Traditionally, every request and response between the service provider and an IoT user has to be routed through the platform provider. Thus, the platform provider can collect information for billing and payments between the related accounts [13]. The centralized authority of an IoT platform provider opens the possibility of a single point of failure. Any errors may interfere with the authentication and payment activities, causing severe damages to the operation and security of the entire system [10]. If a malicious entity (e.g., a hacker or an insider) compromises a platform provider and modifies any transaction, how do they detect the incorrectness of the data? It is imperative that account holders trust their IoT platform provider. There are issues if multiple and federated IoT platforms exist and the service provider and the IoT user are registered at different IoT platform providers [13].

In most financial transactions, the parties involved are assumed to be curious adversaries and are mutually distrustful. This leads to the requirements that are needed to have trust in the transactions. The parties involved must be able to claim and prove the billing and payment to a financial service without any loss of privacy. Fundamentally, the following requirements should be addressed for a trustworthy financial transaction in a less-than-fully-trusted network:

- Accountability
  In an accountable information exchange, the involved parties know how the respective actions will be carried out and who is responsible for the transaction failure because of the system fault [14]. Furthermore, the parties can prove the transactions’ performance in the case of successful execution or failure.
- Reliability
  Basically, the information in the financial statements is reliable if the financial transactions can be checked, reviewed, and verified by the concerned person with objective evidence (e.g., an invoice or a contract). In other words, reliability can be achieved by improving the internal control functions over the financial transactions [15].
- Privacy-preserving
  The data regarding transactions and payment history from financial institutions have historically served as the foundation of most of the financial decision models (e.g., credit scoring) [16]. The availability of historical data and private information across a range of financial transactions is a key factor affecting the users’ privacy concerns. In a privacy-protected financial model, there is no ability to leverage personal data from transaction history, which leads to user identification and profiling.
- Efficiency
  Efficiency means achieving similar levels of impact with fewer resources. Increasing the impact with similar levels of existing resources enhances the effectiveness of a financial model [17]. Data interoperability (at the syntactic or semantic levels) between different financial models enhances the data exchange efficiency. For an IoT ecosystem, an efficient financial system should provide the interoperability capability between different platforms, in which different IoT applications are able to interconnect with each other [18].

While accountability makes the billing and the claimed payment to be provable, the reliability provides a virtual connection between the transaction parties. In this scenario, the third-party entity and IoT consumers can participate in a direct financial contract, in which the IoT platform provider plays no proxy role. The billing and charging functions are expected to provide trustful and efficient services. From this view, the real-time and end-to-end properties helps to bring crucial accuracy and reliability to the charging and billing functions for the consumers and service providers.

In many financial transactions, payment does not require the principals to be identified [13]. By extending such property to other functions, the privacy of the transaction parties is preserved. For instance, having no link to the real identity of the entities involved in a billing and charging system, we can provide a pseudonymity feature that leads to the privacy being preserved. Based on this decoupling, service providers cannot determine whether their service was accessed by a specific consumer. The efficiency property of a billing and charging system indicates the suitability of the scheme to work with different infrastructures without conflicts. For an IoT ecosystem, an efficient billing and charging system should provide the interoperability between different platforms. Unlike centralized systems, in a decentralized user-to-user transaction, all the mentioned requirements need to be achieved without the need for intermediaries or a central regulatory authority. In this scenario, the blockchain can validate any transaction between users.

## 3. Related Work

Privacy-centric blockchain-based applications in the fields of energy trading and IoT ecosystem have attracted significant interest. Because of the distributed nature of blockchain, the transactions’ data available in the shared ledger make blockchain users susceptible to privacy attacks. In a linking attack, an attacker tries to find some facts linked to the user’s private data (e.g., user’s real identity) [19,20]. The attacker tries to characterize sensitive information about a set of individuals and monitors their transactions for a certain period of time. To address this situation, several privacy approaches have been proposed to decouple the users’ pseudonymous identities from the specific transactions they make, thereby preventing attempts to link the transacting parties on the based of the data that appears in the blockchain [21,22].

Ephemeral pseudonyms (e.g., ephemeral wallets in Bitcoin [23]) are used to provide anonymous services. Basically, they are generated to make it difficult for a linking attacker to link together the various transactions recorded in the ledger [10,21]. In such systems, users randomly generate new message addresses for each new transaction [10,20,24,25]. In the solution proposed in [20], a fresh id (used as a key) is used for each transaction. To avoid connecting the pseudonym and a user by matching the energy consumption and the user’s behaviours, in [25], each user generates multiple pseudonyms and submits his power consumption data under different pseudonyms. Despite these pseudonym solutions, the effective heuristics analysis used in the linking attacks has shown the possibility of the re-identification of the users. Meiklejohn et al. [26] identified the Bitcoin parties and the interactions between them by a clustering heuristic based on the change addresses to the cluster addresses belonging to the same user. Furthermore, the ephemeral pseudonym solutions are not practical for financial transactions because of the requirement of long-term identification, and because of the heavy computational burden imposed on IoT users.

Furthermore, a user’s energy consumption data are obfuscated to protect the user’s privacy [27,28,29]. The semantics of energy consumption can be used for user profiling. Abidin et al. [27] attempted to preserve a consumer’s privacy by hiding the details of the energy consumption. By aggregating the private data of the energy usage reported by the smart meters in [27], the energy suppliers compute only the final monthly bill per customer, but not the individual metering data per time slot. Sun et al. [28] hid the household electricity load by using the thermal appliances and energy units. They proposed an opportunistic use of household energy storage units such as electric vehicles (EVs) and heating, ventilating, and air conditioning systems to reduce or eliminate the reliance on local rechargeable batteries for load hiding. An intermediate position between the service provider/aggregator and the consumers is used to blur the energy consumption/generation data. Azar et al. [29] proposed a virtual power plant as an intermediary on behalf of a group of neighborhood prosumers to negotiate with the aggregator, where no private information of the prosumers was shared. Despite the negotiation, the issues related to trustworthiness are possible challenges between prosumers and the compromised aggregator in this approach. In Hy-Bridge, we address the trustworthy issue by using a subnetwork blockchain between consumers, in which all P2P energy transactions are monitored and authorized by the group members. As the bridge is a member of the credit-sharing group and reports the power usage into the main blockchain, its trustworthiness can be monitored by imposing an appropriate policy in the subnetwork blockchain.

Off-chain interaction solutions are used to address the privacy issue with the blockchain by performing several P2P transactions between two parties without writing them into the blockchain. The on/off-chain mechanism enables the deployment of only the on-chain process onto the blockchain. This conserves the resources of the blockchain and hides the sensitive information involved in the off-chain transactions from the public [10,30]. Erdin et al. [31] used an off-chain payment for EV charging stations by building a payment network in parallel to the main ledger, with permissions and signatures to eliminate the high transaction fee and address the privacy exposure problem. Khalil et al. [32] extended the payment channel to a set of users in a payment channel network. As the payment networks, these subnetworks allow payments to be made between parties that are not at the same moment connected by a payment channel. Despite the benefits of the off-chain mechanism to preserve privacy and decrease transaction fees (e.g., in Bitcoin), this method has its limitations, namely limited channel capacity, data privacy in payment transaction routing, and the cost of opening and closing channels [31,33]. By dividing the transactions in two separated blockchains with different purposes, Hy-Bridge does not suffer from the aforementioned limitations.

## 4. Preliminaries

### 4.1. Overview of Blockchain

Blockchain is a decentralized database that records the chain information of the digital events or the public ledger of all the transactions that have been executed and shared among the participating parties [34]. There are two major classes of blockchain systems, namely public and private. In a public blockchain, any node can join and leave the system, whereas, in a private blockchain, there is an access control mechanism to authenticate and authorize the nodes. Thus, in a private blockchain, the identity of each node is known by the other nodes [35]. Activities in a private blockchain are only visible and limited to the authenticated nodes. A hybrid blockchain combines the characteristics of both public and private blockchains and exhibits the characteristics of both with the consensus process being controlled by known, privileged servers. As the copies of the blockchain are only distributed among the entitled participants, the hybrid blockchain is only partly decentralized [36].

**Figure 2:**
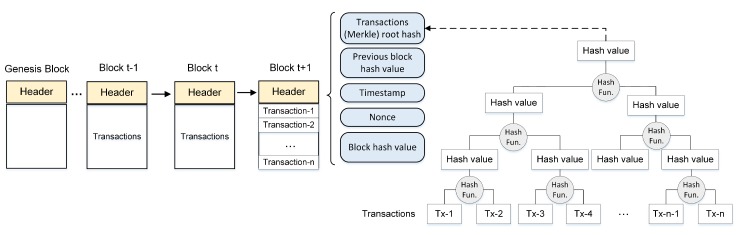
Blockchain structure.

As depicted in Figure 2, each block of a blockchain is composed of a header and a list of transactions. The header consists of the hash value of the previous block, timestamp, nonce, the hash value of all the transactions in the block, and the hash of the state after processing the block. The previous block hash in the header is a hash value that points to the previous block and the timestamp is the current time. The timestamp proves that the data must have existed at the time so were recorded in order to get into the hash [23]. Nonce is a 4-byte random number used to ensure the uniqueness of the hash calculation and prevent any replay attack. To detect any unauthorized data tampering, the blockchain technology has two mechanisms for integrity protection. First, the global states of the chain are protected by a hash (Merkle) tree root of all the transactions in the block [35]. The addition of a new transaction and any state change leading to a new root hash value. A block’s hash value consists of the hash of the block after processing. Second, the block’s history is protected by chain-like linking to the previous block. As shown in Figure 2, a newly appended block contains the hash value of the previous block, thus making the blocks immutable once they are appended [35,37]. This chained hash roots back to an initial block called the *genesis block* generated by a miner.

### 4.2. *k*-Anonymity

In the dataset security, to avoid being identified by linking attacks that combine the data with other publicly available information, Samarati and Sweeney proposed the concept of *k*-anonymity [38]. *k*-Anonymity provides the guarantee that in a set of *k* objects with a similarity, the target object is indistinguishable from the other k−1 objects [39,40,41]. The larger is the value of *k*, the greater is the implied privacy, as the anonymous objects are identifiable with the probability *1/k* [38].

The basic concept of *k*-anonymity is to break the link betweenthe defined identity and the object of the dataset by hiding the information among similar anonymous objects. This process involves the application of the operations of data suppression and value generalization [38,42]. In a suppression operation, some or all the attributes of an object are deleted, and generalization involves replacing specific values of attributes such as age with more general ones (e.g., ranges or intervals). In general, the attributes of a record in the dataset are categorized as identity, quasi-identifier (QI), and sensitivity attributes. While the identity attribute can uniquely identify an individual (e.g., name), QI attributes (e.g., gender or zip code) are more general and can potentially be used for identification. The sensitivity attribute indicates the confidential and sensitive information of an individual (e.g., a disease) [42]. Practically, inference attacks exploit QI attributes, which can be linked to external data, and the background knowledge to identify an object in the anonymity set [43]. To prevent a linkage attack with external data, it needs all *k* individuals in a *k*-anonymity set or equivalence class in a dataset to have similar and indistinguishable QI attributes and if possible sensitive attributes. From this viewpoint, the processes of generalization and suppression change the attributes and generate anonymity sets by masking the original dataset.

#### 4.2.1. Anonymity Degree

Information entropy can be used for an anonymity group to measure the level of anonymity provided by the anonymity process. If we consider each individual in an anonymity model of *X* as an information point, H(x) shows its entropy value. Suppose $p_{i}$ is the probability of identifying the *i*th individual in the anonymity set with *k* members; then:

(1) $$H\left(x\right)=-\sum_{i=1}^{k}p_{i}log_{2}\left(p_{i}\right)$$

The maximum entropy, $H_{M}$, of a *k*-anonymity set is achieved when all of the *k* individuals have the same probability measure of 1/k to be identified:

(2) $$H_{M}=log_{2}\left(k\right)$$

The information that an adversary can achieve with an attack on this anonymity set can be expressed as follows:

(3) $$\frac{H_{M}-H\left(x\right)}{H_{M}}$$

which is normalized by dividing by $H_{M}$ [41]. Thus, the anonymity degree was defined by Diaz et al. [44] as follows:

(4) $$d=1-\frac{H_{M}-H\left(x\right)}{H_{M}}=\frac{H(x)}{H_{M}}$$

An anonymity degree of *d* depicts the anonymizing level of the anonymity model and is a value between 0 and 1 (0≤d≤1). An anonymity model has the minimum value of the anonymity degree (d=0) if an individual in the anonymity set appears to be identified with the probability of p=1, whereas if all of the individuals have the same probability of being identified (p=1/k), the model has the maximum value of the anonymity degree (d=1) [41].

#### 4.2.2. Information Loss

As a side effect of anonymization, the processes of deleting and generalizing result in some information loss. By comparing the masked data to the original one, we can estimate the information loss. The more similar are the data, the less is the information lost [45]. On the basis of the Shannon entropy, in a possible dataset map f:X⟶Y with the probability measures of *p* and *q*, respectively, for the datasets of *X* and *Y*, the information loss associated with the map *f* is defined as the difference between the information entropy in the mapped and the original datasets [46]:

(5) H(p)−H(q)

For instance, in a dataset map of f:{a,b}⟶{c} with the uniform distribution on {a,b}, the probability measures of {a,b} and {c} are p=1/2 and q=1. In this regard, H(p)=ln(2) and H(q)=0. The information loss associated with this map is equal to ln(2). Therefore, we lose one bit of information with the data masked by map *f* [46].

In the data anonymization, the information loss measures how well the masked dataset and the generalized attributes approximate the original ones. The information loss imposed by the masking dataset *D* to $\bar{D}$ in an anonymization process can be measured by using the non-uniform entropy (NE) as follows [45,47]:

(6) $$NE=-\sum_{i=1}^{n}log_{2}Pr\left(D_{i}|{\bar{D}}_{i}\right)$$

where *n* indicates the number of rows (records) in the datasets and Pr is the probability that a particular value in the row $D_{i}$ can be found in the generalized set in $\bar{D_{i}}$. This metric is based on the probability of correctly guessing the original attribute of a record given its generalized data. The calculation of Pr is as follows [47]:

(7) $$Pr\left(a_{m}|b_{m}\right)=\frac{\sum_{j=1}^{r}I\left(D_{j}=a_{m}\right)}{\sum_{j=1}^{r}I\left({\bar{D}}_{j}=b_{m}\right)}$$

where $a_{m}$ is the original attribute value, $b_{m}$ is the generalized value of the attribute, *r* shows the number of attributes in the record, and I() is the indicator function. For a set A⊂Ω, the indicator function of $I_{A}:\Omega ⟶\left\{0,1\right\}$ is a random variable, in which:

(8) $$I_{A}\left(\omega \right)=\left\{\begin{array}{c} 1\;\;\;\;\;\;\;\;if\;\;\;\;\omega \in A\\ 0\;\;\;\;\;\;\;\;if\;\;\;\;\omega \notin A \end{array}\right.$$

In this metric, each cell (attribute) in the dataset is compared to the generalized cells in the anonymized set.

To measure the information loss imposed by suppression, the Kullback–Leibler (KL) divergence provides an objective assessment on the quality of data after the suppression process [48]. Basically, the KL divergence measures the difference between two probability distributions p(x) and q(x) and indicates the information lost when q(x) is used to approximate p(x). The KL divergence is denoted as $D_{KL}\left(p\left(x\right)||q\left(x\right)\right)$ and is measured as follows [49]:

(9) $$D_{KL}\left(p\left(x\right)||q\left(x\right)\right)=\sum_{i=1}^{N}p\left(x_{i}\right)\left[log_{2}p\left(x_{i}\right)-log_{2}q\left(x_{i}\right)\right]$$

The more generic method is as follows:

(10) $$D_{KL}\left(p\left(x\right)||q\left(x\right)\right)=\sum_{i=1}^{N}p\left(x_{i}\right)\left[log_{2}\frac{p(x_{i})}{q(x_{i})}\right]$$

To summarize, greater privacy is commensurate with increased information loss and a decrease in the data utility level. A balance between the level of privacy and the information loss is needed to achieve an appropriate level of data security with the resultant utility of the data (e.g., data analysis) [47].

## 5. Trustful Billing and Charging in IoT Energy and Utility Markets

Hy-Bridge is a hybrid blockchain with subnetworks. The proposed architecture consists of a main blockchain (MaBC) for trustworthy billing and charging transactions and one or more subnetwork blockchains (SuBCs) among the IoT end users for the P2P energy transactions in microgrids. We assumed that each IoT network consisted of some IoT devices and was connected, via a gateway, to the other entities in the other layers. We considered two types of transactions in our model, namely the user-to-user (U2U) transactions and the user-to-server (U2S) transactions. The U2U transactions were carried out among the IoT devices of one user or among those of different users. The U2S transactions, such as billing and charging transactions, were carried out among the IoT users and the entities in the upper layers (e.g., the platform layer or the enterprise layer).

**Figure 3:**
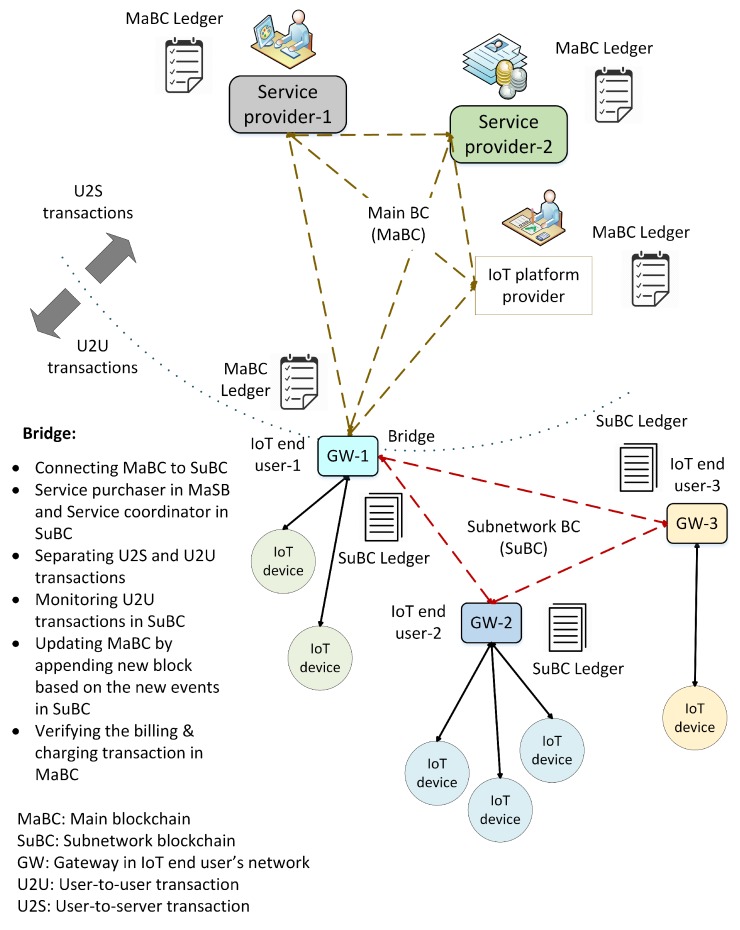
Proposed hybrid blockchain consists of a main blockchain for billing and charging transactions and subnetwork blockchains for P2P transactions.

Figure 3 shows the proposed hybrid blockchain for the IoT energy and utility market. In this architecture, we envisaged a device in each IoT network acting as a control center, which could be a separate device or a part of the gateway. For the sake of convenience, we considered a gateway to be the control center. It was a miner and processed the incoming and outgoing transactions. In other words, the gateway was a blockchain node and collected the transactions into a block and appended this block to the chain. It used local data storage and performed all functions, such as authentication, authorization, generation of genesis transactions, and key management. IoT devices could communicate directly with one another in the local network or with an external entity through the gateway.

When a new service is purchased, a blockchain is established between an IoT user (the property manager) as the consumer, the service provider(s), and an IoT platform provider, as Hy-Bridge is a service-based system. Each user is identified by a unique *client-id*, as its public key, that has no relation to its real identification. A genesis transaction is generated by the service provider when a new service is purchased by an IoT user. Any transactions related to the service, such as purchasing credits, utilization, updating, charging, and billing, are packed into the block after having been verified by a miner. Depending on the service type, e.g., roaming, more service providers can join the blockchain. For instance, if a service can be provided by a provider in addition to the ones already in the group, they can join the blockchain and copy the ledger. In this regard, service level agreements (SLAs) among the service providers are required to define the service policies. When a user registered with service provider *A* requests credit offered by service provider *B*, Hy-Bridge checks an SLA between these providers from the platform or platforms. If there is an SLA for this transaction, a U2U transaction is created. The transaction is first shared among SuBC(s) and then a U2S transaction is propagated to provider groups in MaBC. The access permission is granted after verifying in the MaBC and the user receives a credit access grant transaction. The credit amount and usage are monitored in both MaBC and SuBC blockchains to detect any malicious or cheating activity.

In the microgrid, the IoT end users establish an SuBC for their P2P energy transactions, such as sharing the remaining energy to the neighbors or access to a shared credit. To set it up, end-users establish a local private blockchain and agree on how to share the energy transactions of their IoT devices. The connections between IoT users can be direct connections between the IoT devices or via an IoT platform provider, depending on their geographical location. The keys required for cryptographic purposes are shared between the IoT gateways. Here, we introduce an interconnection position called the *bridge*, connecting the MaBC and the SuBC(s). The gateway of the IoT user who purchased the service and shares it with the other users is considered to be the bridge.

### 5.1. Bridge

The property manager, who purchases the energy service and shares it with the other users in a credit-sharing group plays an interface role in the proposed hybrid blockchain. As this user is simultaneously a member of the MaBC and the SuBC(s), he/she is called the bridge. The bridge is a wholesale service buyer in the MaBC and acts as a retailer and service coordinator in the SuBC(s). By separating the transactions in the subnetwork from those outside, the bridge protects the IoT users’ privacy as an anonymizer. To this end, the bridge implements *k*-anonymity and performs the anonymization processes of generalization and data suppression to eliminate any links to the end users’ identities. Thus, the P2P energy transactions between the IoT devices cannot be monitored by the upper layer’s entities in MaBC. The bridge generates the corresponding transactions and appends them to the MaBC based on the activities of users in the SuBC. Because of the data anonymization, these transactions do not contain any private information about the user (e.g., IoT user and/or IoT device). The bridge acts as an interpreter between the MaBC and the SuBC(s). The bridge updates the local subnetwork by generating an U2U transaction when there is a new transaction in MaBC, such as service updating or billing and charging.

### 5.2. Credit-sharing Group

We now expand upon the fact that paying for the service does not require the identity of the principals [13] to introduce a new feature. The IoT users can create a credit-sharing group and share their service access rights with each other. The credentials, connectivity, security features, or the access rights to a service purchased by an IoT user account are available to the others in the group. The access conditions (e.g., IoT device type, access time, and duration) are manageable by the group members. The IoT users only need to join an SuBC and distribute a ledger to monitor the credit usage to create a credit-sharing group.

Group members follow some policies or rules and monitor the registered service state and thus manage the credits. The rules would depend upon the IoT users’ agreements between themselves, and the current status of these agreements. Monitoring is performed by a smart contract through the SuBC. Smart driving assistance, P2P energy transactions, electric vehicle charging, and mobile wallet services are some possible applications for this model. For instance, a smart apartment building might have an array of solar panels, a Tesla Powerpack, and a large solar-heated hot water tank—all owned by the building’s owner. The batteries and hot water tank(s) would be topped up from the public grid during the off-peak hours when the electricity rates were the lowest. The power purchased in the landlord’s name is accessible by the members of the credit group via the P2P energy transactions. The IoT devices of the members jointly access the power on the microgrid. All use is logged in the SuBC with an U2U transaction. The bridge, in contrast, who purchased the service, shares the related events in MaBC by an U2S transaction with the upper layer entities (e.g., a service provider and an IoT platform provider). The U2S transactions cover all of the events related to the purchased service, such as access events, offering new features, updating, and billing and charging.

### 5.3. Local Block

**Figure 4:**
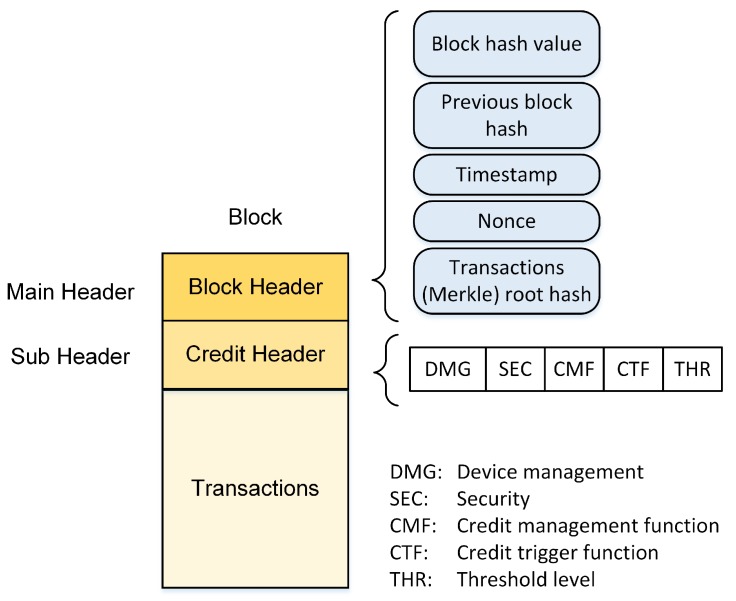
Local block structure for U2U transactions in SuBC.

Local blocks are introduced to handle the U2U transactions in SuBC. Each local block has two headers, a block header and a credit header. The credit header is used to authorize devices and enforce the credit-sharing policy. As shown in Figure 4, it has five parameters. Device management (DMG) refers to the device making the transaction. For local devices, this parameter is used as the *Device-id* to distinguish the IoT devices allowed to access the service. The security parameters required for service access, such as identity management and access control, are in the security (SEC) field. The credit management function (CMF) parameter has the negotiated policies required for credit management. The IoT users abide by these policies for service access, duration, amount, and priority. The total amount of credits spent is measured and shown by the credit trigger function (CTF) parameter. The threshold (THR) parameter shows the utilization threshold; when the usage reaches this threshold, access to the service is available only for the predefined high priority IoT devices.

In each IoT user network, the gateway generates a genesis transaction to add a new IoT device and shares a key with this device. A shared key is allocated to the devices that are eligible for utilizing a shared service, according to the access control list. IoT devices that are allowed to access the shared service are introduced by an IoT user to set the policies. The policies negotiated between the IoT users are used to control access. The access time, duration, and consumption limits per device are explained by these policies.

## 6. Use Cases and Possible Applications

Since Hy-Bridge is a service layer framework, it is general purpose and there are several applications that can benefit from its features. In this section. we introduce three possible Hy-Bridge use cases.

### 6.1. Energy and Utilities

Hy-Bridge is capable of providing transaction security and reliability for access management, smart meter control, and power consumption in the IoT energy and utility market. Events in the microgrid and P2P power transactions are indicated by the U2U transactions in the local blocks of the SuBC. In the local block (SuBC), DMG shows the IoT devices allowed access to the solar power and the access conditions are indicated by the CMF field. To access the service, an IoT device sends a power access request. On the basis of the information in the DMG, the access is granted. The smart meter at each IoT user’s site measures the power usage and the CTF field is updated by the IoT user’s gateway. When the threshold level is reached, as shown by the THR field, power access is stopped by the IoT gateway. In the MaBC, by appending a new block, the bridge distributes the power service usage to the other entities. Therefore, the P2P energy transactions are distributed in the SuBC and the billing and charging transactions are distributed in the MaBC.

### 6.2. Electric Vehicle Charging

EVs are bringing new perspectives for power consumption to the smart grid [50]. Trust and privacy challenges with the vehicle-to-grid (V2G) data create new challenges in smart grid data acquisition and management. Electric vehicle charging (EVC) is a new field of coexistence for IoT and blockchain technologies. Several applications have been introduced for smart contract and machine-to-machine (M2M) connections for pole-charging EVs. Path traffic query, autonomous charging, station selection, billing, and automated payment are the possible applications for EVC. For example, Blockcharge [51] provides a mechanism for blockchain-based charging, authentication, and billing for EVs. In Blockcharge, the billing and payments are done automatically via the use of a cryptocurrency (Ethereum blockchain).

We can provide a new service for EVC applications with our credit-sharing feature. A hybrid blockchain architecture can be established when a prepaid/postpaid vehicle charging credit is purchased and an SuBC is established to create a credit-sharing group to provide a trusted and private transaction network. With this credit-sharing group, it is possible to create a *k*-anonymity set for some EVs (or owners) and anonymize their information in the M2M transactions. In this hybrid architecture, the bridge connects the MaBC to the SuBC and is responsible for the billing and charging transactions. Furthermore, *k*-anonymity is preserved in the SuBC, where EVs share the credit for charging among themselves, and the billing and charging services are handled in the MaBC. EVChain [33] is an accountable and trustful solution for the EVC market to preserve the EV owners’ privacy on the basis of our introduced credit-sharing feature.

### 6.3. Credit Transfer

Mobile wallets are common services provided by telecommunication companies or non-banking institutions, used for e-commerce, bill payment, and other financial transactions. They are desirable by customers and service providers as they offer ubiquitous payment facilities, digital vouchers and coupons, and location-based services and support different payment sourcing types [52]. By incorporating multiple levels of authentication, mobile wallets are protected against security threats. Exploiting the blockchain technology helps to cope with the reliability and trust issues in digital wallet services. A customer-to-customer credit transfer service (e.g., Etisalat wallet [53] allows a customer to transfer a balance from his/her wallet to others in the same or different network. Normally, transfers are centralized and handled by the service provider. Hy-Bridge can provide a decentralized and distributed architecture for credit transfer. In this architecture, a customer shares the credit with others in the SuBC and sets the required policies. The balance, usage conditions (e.g., time and threshold levels), and priority are controlled in the SuBC. Transactions that lead to balance updates or credit charging are distributed in the MaBC between the bridge and the service provider. In this case, Hy-Bridges helps to omit the central role of the wallet service provider and customers securely share their credit with other customers in a credit-sharing group.

## 7. Implementation

As mentioned above, we defined an energy use case scenario and considered a smart building with a local energy source of rooftop solar power system. The solar power is assumed to be offered by a service provider and the property manager resells power to the tenants through a microgrid. The tenants are IoT users who access the power service through a credit-sharing group. To implement the proposed hybrid blockchain for this scenario, we used and developed the Python blockchain package provided by Eric Alcaide (University of Barcelona, Barcelona, Spain), and available on GitHub [54]. We implemented the bridge node that interconnects the MaBC to the SuBC(s). We added a SuBC in which the power consumption and the P2P power transactions in the credit-sharing group, indicated by a U2U transaction, are reflected in the MaBC by the bridge node. As the main focus of this implementation was to provide trustful transactions and a hashed ledger, the miner and mining process were not included. Therefore, in this implementation, the processes of credit-sharing group creation, U2U and U2S transaction generation, block hashing, linking, validating, and ledger entry in both the blockchains were implemented. Furthermore, the bridge roles of data anonymizing, connecting and updating both blockchains were implemented and evaluated.

### 7.1. Setting Power Sharing Group

As shown in Figure 3, the bridge simultaneously joins the MaBC and the SuBC to share the power and create a credit-sharing group. To initiate the group, the credit header in the local block is initialized. At this point, all of the permitted IoT devices are registered in the DMG field, the SEC field has any required keys, the CMF shows the current negotiated policy, the CTF shows the total power use (in kilowatt hours), and THR indicates the power threshold level (in kilowatt hours). This information is packed in the genesis block in the SuBC. In this use case scenario of Hy-Bridge, the landlord of the smart building is the bridge and connects the SuBC to the MaBC.

### 7.2. Transaction Handling

**Figure 5:**
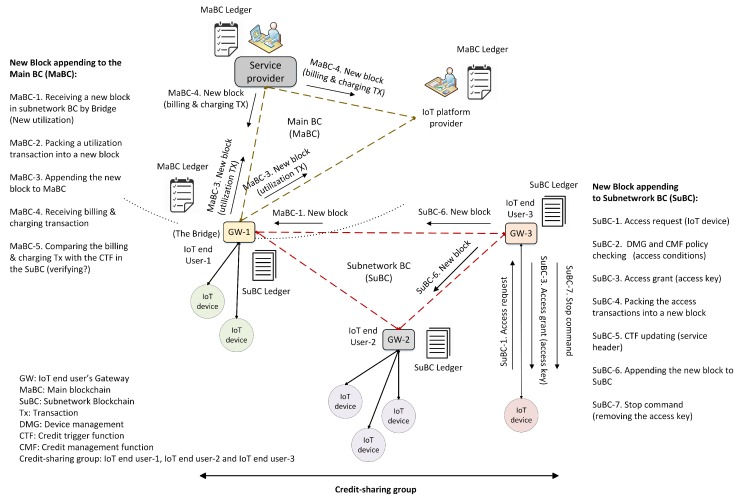
Transactions handling. MaBC-x, transaction progress in MaBC; SuBC-x, transaction progress in SuBC.

Figure 5 shows the steps of accessing power in a credit-sharing group and how billing and charging transactions are handled. To start, an IoT device sends an access request to its gateway and requests permission to use power. The gateway checks the credit header to verify the eligibility of the IoT device and grant permission. Based on the DMG parameter, the gateway either rejects the request or grants access by allocating an access key. Before allocating the key, the gateway checks the CMF’s policies with respect to access conditions (e.g., access time and duration) and then shares the key with the device. The service is accessible as long as the allocated key is valid. Thereafter, the gateway packs these access transactions into a new block and updates its header by changing the credit amount in the CTF field. Finally, the new block is appended into the SuBC and shared with the other nodes.

**Figure 6:**
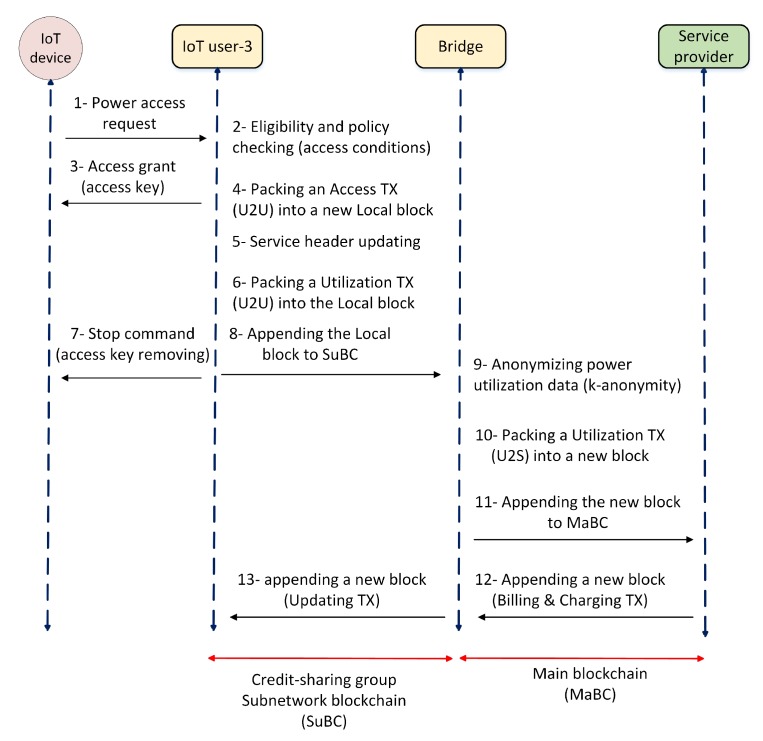
Power access and utilization transaction handling.

As all the nodes in a private blockchain need to participate in the consensus [55], the new block added to SuBC has to be checked and verified by all the IoT users. Any power consumption leads to the creation of the P2P energy transactions and appending a new U2U block to SuBC. As shown in Figure 6, the bridge anonymizes the power consumption data and generates a U2S transaction to append onto the MaBC by detecting a local block in the SuBC. In the MaBC, the service provider regularly issues a billing and charging transaction to a client-id by appending a new block. As the updated service use is available to the bridge in the SuBC, by receiving a billing and charging transaction, the bridge can verify and confirm the issued bill.

**Figure 7:**
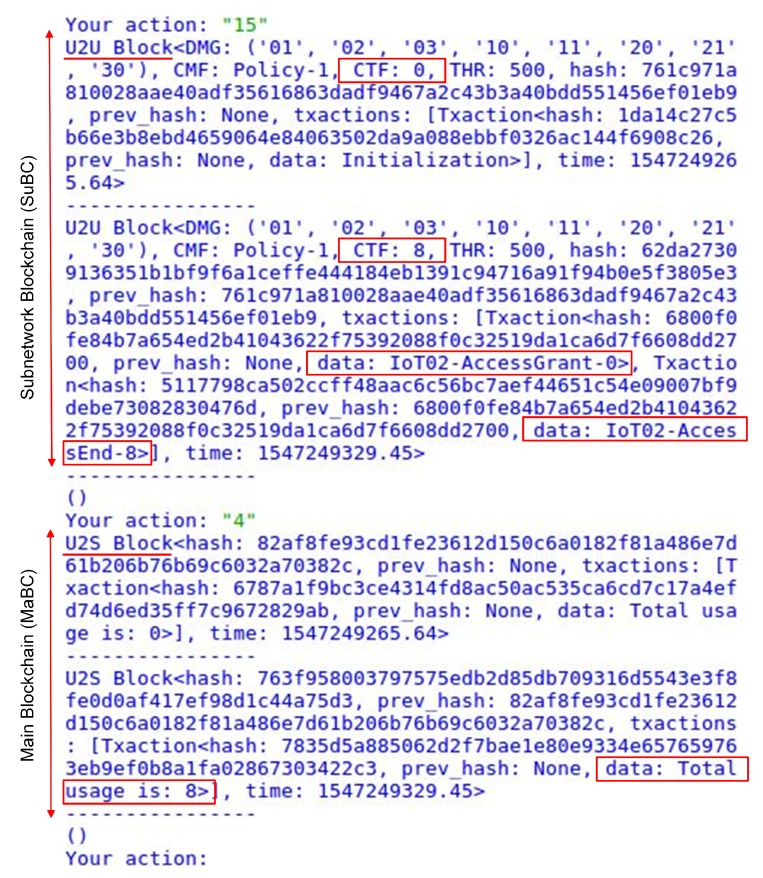
Energy usage transactions in SuBC and MaBC.

Figure 7 shows the blocks that were created in the scenario shown in Figure 5. In the two U2U blocks (in SuBC), the credit header fields show the information about the credit-sharing group, such as registered IoT devices in the DMG, the policy used, the power consumption, and predefined threshold. The second U2U block consists of two P2P power transactions that indicate the start and the end of the power consumed by an IoT device (*IoT02*). The CFT field in the credit header shows the amount of power consumed by the IoT device (CFT = 8). Simultaneously, by appending a U2U block to the SuBC, a U2S block is appended to the MaBC by the bridge to show the power consumption. Although a U2U block contains details about the service access, such as registered IoT devices, the IoT device that used the power, amount consumed, and the time of access, no further data (e.g., IoT user and IoT device) are reflected in the U2S blocks by the bridge. In fact, the bridge anonymizes the power consumption data and reflects no details related to the IoT users of the credit-sharing group to the upper-layer entities in the MaBC. The details of the anonymization process are described and analyzed in the next section.

## 8. Trustworthiness and Privacy Analysis

In a linking attack, an attacker tries to find some facts linked to the user’s real identity [19,20]. The attacker tries to characterize sensitive information about a set of individuals and monitors their transactions for a period of time. To address the privacy challenges, we exploited the *k*-anonymity solution in the credit-sharing group to conceal IoT users’ real identities in the power consumption data. In this section, we analyze the trustworthiness and the privacy preservation provided by Hy-Bridge.

### 8.1. Trustworthiness

The decentralized nature of the blockchain allows one to omit the intermediate role of the IoT platform provider in the policy-making and billing platforms. Through this feature, we provide a trustable workflow for the billing and charging services in a trustless network. Transactions are distributively logged and verifiable. This trustful end-to-end functionality addresses the accountability and reliability requirements to have trustful financial transactions between the IoT users and the service providers.

In the Hy-Bridge, an IoT user’s local activities are kept private. The details of the activities of the IoT devices are hidden from any unrelated entity because of the separation between the U2U and the U2S transactions because of the bridge. In brief, the bridge conceals the local transactions from the upper-layer entities and updates the CMF by purchasing new credits. From the viewpoint of its trustworthiness, the blockchain technology enables the bridge able to detect financial frauds and misuses. As the updated service use is available to the bridge in the SuBC, by receiving a billing and charging transaction in the MaBC, the bridge can verify the issued bill.

In contrast, in the SuBC, the shared service is accessible by the authenticated IoT users in the credit-sharing group. In this group, only the transactions related to the shared service (e.g., credit updating and service access) are shared with the trusted nodes in this private blockchain. The other local transactions are not shared with the group. As such, there is no privacy violation for an IoT user’s other activities. Any access to the shared service is based on the agreed policies and is monitored by all group members in the SuBC. Since SuBC is a private blockchain network, it is limited to its authorized members, which can store the private ledger [56]. All information about the credit, consumption, and access policies are available. Each credit access (e.g., power consumption) leads to update CTF in the credit header that needs to be verified by SuBC members. Each member of the credit-sharing group can trace with recovery options in an event of fraud or malicious activity through the credit header in the local block. Proper policies can be negotiated and assigned to punish a cheating member. These policies are available along with the credit management policies by the CMF parameter.

### 8.2. Privacy Preservation

By separating the P2P energy transactions in the SuBC from the billing and charging transactions in the MaBC, we attempt to conceal the IoT users’ activities with the energy transactions in the microgrid. In the credit-sharing group, the information on the power usage of the IoT users in the microgrid is saved in a table. This information is available as the P2P energy transactions in the SuBC. Table 1 shows an example of the IoT device power consumption in a microgrid for one day. This microgrid consists of four IoT users that access the subnetwork power grid in the smart building. In this table, the user’s identity (*IoT-usr*), device identity (*IoT- dvc*), user’s unit number in the building (*Unit*), the power used in watt hours (*Usage*), IoT device type (*IoT-type*), and the time and duration (*Time/Dur*) are registered for each power consumption.

To preserve IoT users’ privacy, the bridge anonymizes the power usage table in the credit-sharing group and generates a new table to be shared with the service group members in the MaBC. In fact, the data in the new table of power usage are a transformed version of the main table in the microgrid. The information in this table is available through the U2S transactions in the MaBC. Table 2 shows the power usage information in the service group and is an anonymous view corresponding to the information of Table 1. By assuming that the number of IoT users in the smart building is available for the service group members (e.g., service provider) by linking to external data, in Table 2, we present a 4-anonymity view of Table 1.

The data in Table 2 are anonymized by the generalization and suppression processes. In these processes, the attributes of the IoT user’s identity and their unit numbers are generalized into the client id (*User*) and address (*Address*). As the IoT device identity links to identify the IoT user, this attribute is suppressed in the new table. The other attributes are not changed and provide information about the power consumption for a registered client id (*User*). The last attribute (*IoT-type*), which shows sensitive information, is considered as the sensitivity attribute and the rest of the attributes are QI attributes for each record in this 4-anonymity set. Individually, there is no information about the IoT users and all of the consumption data are related to the client id, which was registered at the time of service purchase. Therefore, the IoT users’ privacy is preserved by concealing their activities by the bridge in the service group.

#### 8.2.1. Anonymity Degree

To measure the anonymity provided by our solution, we used the anonymity degree introduced in Section 4. We assumed that, on the basis of side knowledge, the attacker knows the number of IoT users in the microgrid. It could be deduced by finding a link between an IoT user and his/her IoT devices registered with another service. The IoT users’ identities are unknown to the attacker and there is no link between the IoT devices and the users in the anonymity table.

According to Table 2, under the best anonymized condition, in which the attacker cannot link an IoT device type to a particular user, the probability to identify an IoT user is 1/4. In the created anonymity set, the anonymity degree is equal to the following:

(11) $$d=\frac{H(x)}{H_{M}}=\frac{-\sum_{i=1}^{4}p_{i}log_{2}p_{i}}{log_{2}\left(4\right)}$$

**Figure 8:**
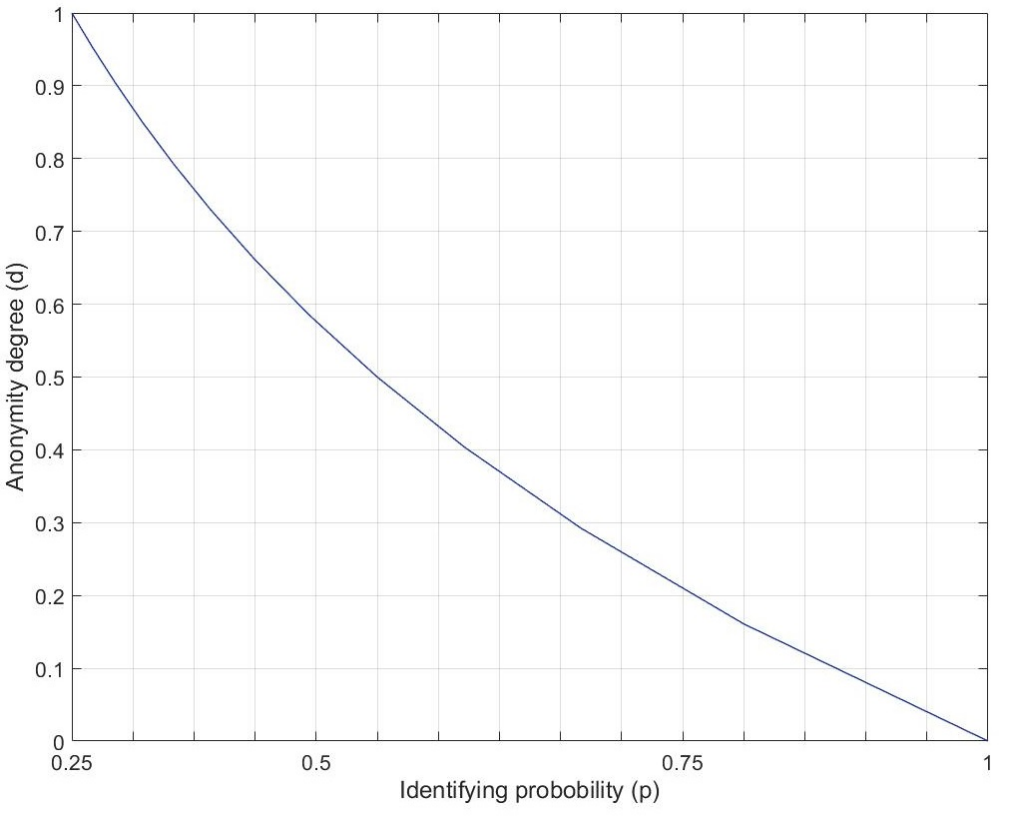
Anonymity degree curve for anonymity set in Table 2.

In Table 2, the maximum anonymity degree (d=1) is achieved by hiding the identities of the IoT users and devices. If the attacker finds an IoT user on the basis of the sensitivity attribute in the table (IoT device type), the anonymity set is shrunk to a 3-anonymity set and the attacker has a probability of p=1/3 of identifying other users. Figure 8 depicts the curve of the anonymity degree for the anonymity set considered in Table 2. The anonymity set has the minimum anonymity degree (d=0) if the attacker identifies the IoT users in a linking attack, on the basis of the side information of the IQ and sensitivity attributes. For instance, by profiling the user’s presence schedule in the building, through a linking attack, the link between the IoT user and the IoT devices can be detected.

#### 8.2.2. Information Loss

To anonymize the power consumption data presented in Table 1 in Table 2, the two attributes of *User* and *Address* are generalized. Furthermore, the *IoT-dvc* attribute is suppressed to avoid any link to identify the IoT users in the credit-sharing group. To measure the information loss imposed by the generalization, the non-uniform entropy introduced in Section 4 is used. As, in each record, two attributes are generalized, the Pr value for each record can be calculated as follows:

(12) $$Pr\left(a_{i}|b_{i}\right)=\frac{\sum_{j=1}^{5}I\left(D\left(j\right)=a_{i}\right)}{\sum_{j=1}^{5}I\left(\bar{D}\left(j\right)=b_{i}\right)}=1$$

Note that the suppressed attribute (*IoT-dvc*) is not considered when measuring Pr. Therefore, the information loss imposed by the generalization for all the records is equal to the following:

(13) $$NE=-\sum_{i=1}^{13}log_{2}\left(\frac{\sum_{j=1}^{5}I\left(D_{i}\left(j\right)=a_{i}\right)}{\sum_{j=1}^{5}I\left({\bar{D}}_{i}\left(j\right)=b_{i}\right)}\right)=0$$

Therefore, the generalization process does not impose any information loss in our anonymization model.

In contrast, suppressing an attribute in the anonymized table does cause information loss. Although the suppression looks rough, it is very useful in practice by hiding information effectively without affecting the values from the irrelevant data [48]. To measure the information loss imposed by suppression, we used the measurement of the KL divergence introduced in Section 4. To anonymize the information in Table 1, the attribute of *IoT-dvc* is completely suppressed. Therefore, no information of this attribute is available in Table 2. Thus, the information loss is equal to the following:

(14) $$D_{KL}\left(p\left(x\right)||q\left(x\right)\right)=\sum_{i=1}^{13}p\left(x_{i}\right)\left[log_{2}p\left(x_{i}\right)-log_{2}q\left(x_{i}\right)\right]$$

As the *IoT-dvc* is removed and not available in Table 2, $log_{2}q\left(x\right)$ is not measurable and the attribute information is lost. Therefore, the information loss of this suppression is equal to the entropy of the *IoT-dvc* attribute given in Table 1. This inference satisfies the concept of information loss based on the Shannon entropy shown by Equation (5). Therefore, the information loss imposed by suppression is as follows:

(15) $$|D_{KL}\left(p\left(x\right)||q\left(x\right))\right|=H\left(p\right)$$

For the example of power usage shown in Table 1, the information lost while suppressing the *IoT-dvc* attribute in Table 2 is almost 3 bits.

Although attribute suppression inevitably leads to information loss, there is no such effect on the data utility in the anonymized information. As the attributes of *Usage (W-hr)* and *IoT-type* remain unchanged, the information presented in Table 2 has the same data utility as that of Table 1.

### 8.3. Performance Evaluation

**Figure 9:**
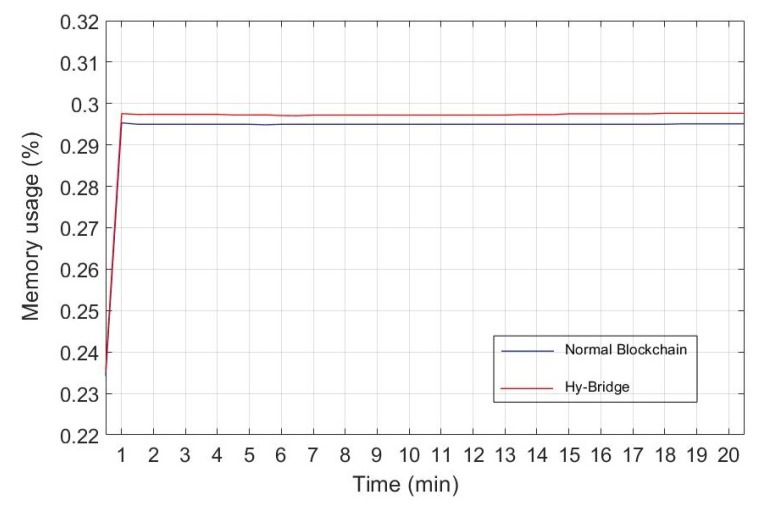
Memory percentageused by Hy-Bridge and the normal blockchain based on IoT power consumption presented in Table 1.

**Figure 10:**
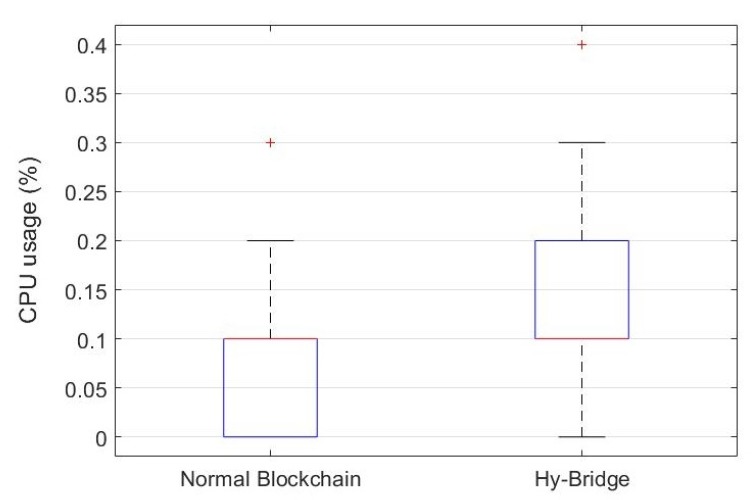
CPU percentage used by Hy-Bridge and the normal blockchain based on IoT power consumption presented in Table 1.

**Figure 11:**
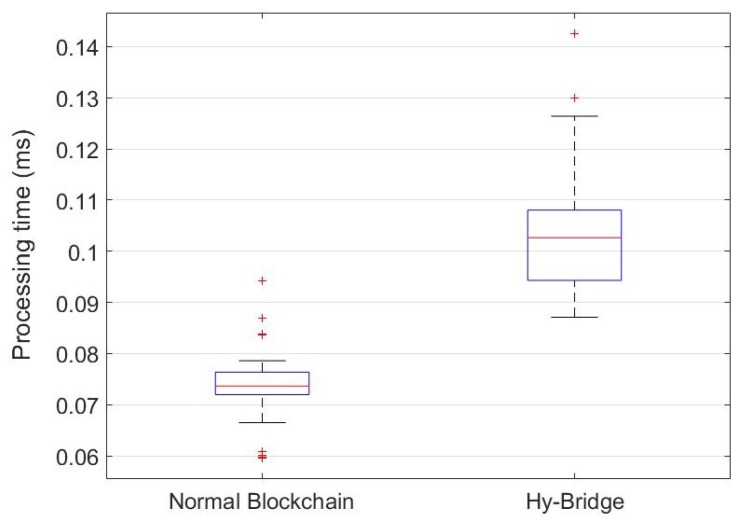
Processing time for block adding to the normal blockchain and Hy-Bridge based on IoT power consumption presented in Table 1.

**Figure 12:**
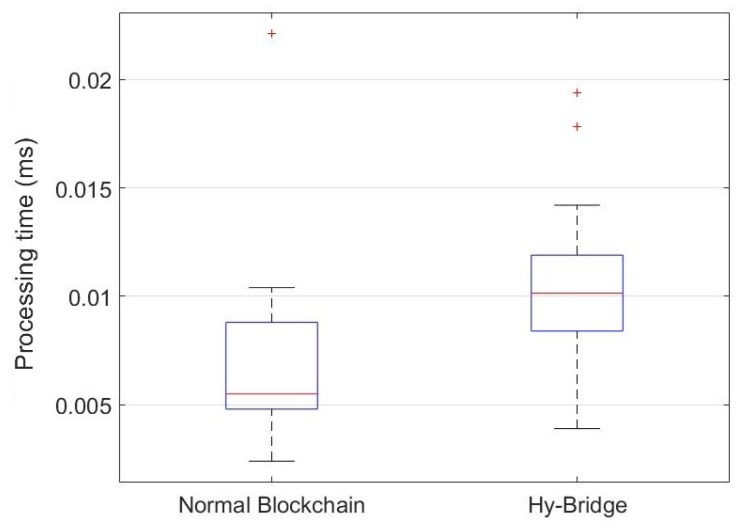
Processing time for block validating in the normal blockchain and Hy-Bridge based on IoT power consumption presented in Table 1.

To provide a practical comparison, we measured the resource (memory and CPU) usage of Hy-Bridge and compared it to a standard blockchain memory and CPU usage. Furthermore, we measured the time processing for block adding and block validating processes in both blockchains. To this end, we simulated Hy-Bridge and the blockchain provided by E. Alcaide [54], which was used to develop Hy-Bridge. The results are based on the IoT device energy consumption scenario shown in Table 1. The memory percentage and the CPU percentage used by Hy-Bridge and the normal blockchain in the same power usage scenario are, respectively, depicted in Figure 9 and Figure 10. The time processing for block adding and block validating processes in the normal blockchain and the Hy-Bridge are depicted in Figure 11 and Figure 12.

Table 3 compares the average processing time and the average usage of the resources required to handle the transactions of the IoT power consumption in the normal blockchain and the Hy-Bridge. According to these results, the normal blockchain on average used 0.293% of the memory and the Hy-Bridge used 0.296%. Based on this, there is no tangible memory usage overhead with the Hy-Bridge. In the view of CPU usage, the Hy-Bridge on average used 0.144%, which is relatively 51% more than the CPU percentage used by the normal blockchain with 0.095% CPU average usage. In the view of time, Hy-Bridge on average takes more time for block handling in comparison to the normal blockchain. While the normal blockchain on average takes 0.074 ms for block adding to the blockchain and 0.007 ms for block validating, the Hy-Bridge on average takes 0.104 ms and 0.010 ms, respectively, for block adding and block validating.

While in the normal blockchain the blocks of power transactions are directly appended, we have more processes in Hy-Bridge to append the P2P power transactions blocks. The effects of those additional processes (e.g., adding credit header and anonymizing) have been shown by the time processing difference for block handling in two blockchains. In Hy-Bridge, the power consumption data of the IoT devices, generated in the SuBC, are indirectly appended to the MaBC. The bridge anonymizes the power consumption data available in the local block and appends a new block to the MaBC. While there was no considerable difference between the memory percentage used by Hy-Bridge and that used by the normal blockchain, the main differences were in the CPU usage and the processing time for block handling. Performing the additional processes (e.g., anonymizing and handling two blockchains) in Hy-Bridge leads to overheads in CPU usage and processing time in comparison to the normal blockchain. Despite those overheads, the efficiency of the blockchain has not been affected.

## 9. Conclusions

In this article, we propose Hy-Bridge, a novel structure for trustful financial transactions in the IoT energy and utility markets based on the blockchain technology. Hy-Bridge is a hybrid blockchain with local subnetworks to isolate the P2P transactions of the IoT users. This architecture provides accountable and trustful transactions in a less-than-trusted network. The accountability and reliability achieved by blockchain provide assurance for the IoT players. By eliminating the intermediate role of the IoT platform provider, the billing and charging transactions are clearly provable and any fraud or financial misuse is prevented by the blockchain technology.

We introduce the bridge position to the isolated P2P transactions and hide the IoT user’s activities in the hybrid architecture of the blockchain. The bridge performs *k*-anonymity protection to preserve IoT users’ privacy in a credit-sharing feature. With this feature, the IoT users in a credit-sharing group anonymously access a shared service. Therefore, the information of the IoT users’ P2P energy transactions in the microgrid is anonymized by the bridge and no private information is leaked to the main power grid. We showed that this anonymization model provides privacy protection with acceptable information loss and data utility.

The concept of a local block with an additional header is introduced to handle the P2P transactions of the credit-sharing group in the subnetwork blockchain. To address the efficiency requirement, the credit header fields are defined in the service layer and the IoT users can manage the credit-sharing with different types of IoT devices. We showed the processing time for block handling and the overall memory and CPU usage imposed by Hy-Bridge in comparison to the normal blockchain. In the future, we will implement Hy-Bridge in some real-world use cases and add the mining process of the blockchain technology to provide practical feedback.

## Figures and Tables

**Table 1:** Power consumption data in credit-sharing group (SuBC).

Time/Dur	User	IoT-dvc	Address	Usage (W-hr)	IoT-type
13:00/20	U-2	21	201	200	Heater
14:00/10	U-1	12	101	120	Vacuum
14:10/30	U-1	11	101	50	Lighting
14:20/60	U-3	31	301	80	TV
15:30/10	U-1	11	101	10	Lighting
16:20/30	U-2	21	201	500	Heater
17:10/20	U-3	30	301	40	Lighting
18:00/240	U-1	11	101	400	Lighting
18:05/120	U-1	10	101	90	TV
18:10/300	U-2	20	201	500	Lighting
18:10/185	U-2	22	201	185	TV
19:00/125	U-3	31	301	100	TV
19:45/180	U-4	40	401	260	Lighting

**Table 2:** Anonymized power consumption data in service group (MaBC).

Time/Dur	User	Address	Usage (W-hr)	IoT-type
13:00/20	S-11	780-Mont St.	200	Heater
14:00/10	S-11	780-Mont St.	120	Vacuum
14:10/30	S-11	780-Mont St.	50	Lighting
14:20/60	S-11	780-Mont St.	80	TV
15:30/10	S-11	780-Mont St.	10	Lighting
16:20/30	S-11	780-Mont St.	500	Heater
17:10/20	S-11	780-Mont St.	40	Lighting
18:00/240	S-11	780-Mont St.	400	Lighting
18:05/120	S-11	780-Mont St.	90	TV
18:10/300	S-11	780-Mont St.	500	Lighting
18:15/185	S-11	780-Mont St.	185	TV
19:00/125	S-11	780-Mont St.	100	TV
19:45/180	S-11	780-Mont St.	260	Lighting

**Table 3:** Average resource usage and processing time to handle the transactions in Hy-Bridge and the normal blockchain.

Resource Usage and Processing Time	Normal Blockchain	Hy-Bridge
Memory usage	0.293%	0.296%
CPU usage	0.95%	0.144%
Block adding time	0.074 ms	0.104 ms
Block validating time	0.007 ms	0.010 ms
